# Clinical value of blood markers to assess the severity of coronavirus disease 2019

**DOI:** 10.1186/s12879-021-06623-5

**Published:** 2021-09-06

**Authors:** Liu-Niu Xiao, Xiao Ran, Yan-Xia Zhong, Shu-Sheng Li

**Affiliations:** 1 Department of Intensive Care Unit of Tongji Hospital Affiliated to Tongji Medical College of Huazhong University of Science and Technology, No. 1095 Jiefang Avenue, Qiaokou District, Wuhan, 430030 Hubei China; 2 Tongji Medical College of Huazhonng University of Science and Technology, Wuhan, 430030 Hubei China

**Keywords:** COVID-19, IL-6, IL-8, CRP, Platelet

## Abstract

**Background:**

Severe acute respiratory syndrome coronavirus type 2 (SARS-CoV-2) is threatening the world with the symptoms of seasonal influenza. This study was conducted to investigate the patient characteristics and clinical value of blood markers to assess the severity of coronavirus disease 2019 (COVID-19).

**Methods:**

187 patients, diagnosed with COVID-19 (non-severe and severe cases) and admitted to hospital between January 27th and March 8th of 2020, were enrolled in the present study.

**Results:**

A higher proportion of clinical symptoms, including cough, expectoration, myalgia, and fatigue were observed in the non-severe group. The level of white blood cell count, neutrophils, CRP, IL-6 and IL-8 were significantly increased, while the platelet count was remarkedly decreased in the severe group. The risk model based on lymphocyte, IL-6, IL-8, CRP and platelet counts had the highest area under the receiver operator characteristic curve (AUROC). The baseline of IL-6, IL-8 and CRP was positively correlated with other parameters except in the cases of lymphocyte, hemoglobin and platelet counts. The baseline of the platelet count was negatively correlated with other parameters except in the lymphocyte and hemoglobin counts. Additionally, there was no connection between the severity of COVID-19 and cultures of blood, sputum or catheter secretion.

**Conclusions:**

The present study suggested that high leucocyte and low platelets counts were independent predictive markers of the severity of COVID-19.

## Background

The coronavirus disease in China, that began in December 2019 (COVID-19), is an emerging lethal respiratory disease and has threatened global health [1, 2]. Based on current epidemiological investigation, over 2 million people worldwide have been affected by COVID-19 [3], which has shown to excess the outbreak of severe acute respiratory syndrome (SARS) in 2002 and middle east respiratory syndrome (MERS) in 2012 [7], undermining the global economy, and destabilizing societies across the world. Full-genome sequencing has labeled the pathogen as severe acute respiratory syndrome coronavirus 2 (SARS-CoV-2), which is a novel coronavirus declared by the World Health Organization (WHO) [3]. SARS-CoV-2 is a type of single-strand positive RNA virus and its genetic characteristics are significantly different from those of SARS-CoV or MERS-CoV [4]. It is reported that the spread from person-to-person in the hospital and community settings has been accumulating all over the world [5]. Since the transmission routes include droplet, contact, and aerosol transmission, quarantine has been an effective method to protect susceptible people and reduce transmission [6].

A total of 90,877 cases and 4744 deaths have been reported in China, moreover, 68,139 cases and 4512 deaths have been reported in the most serious region of Wuhan by May 20, 2020. The 28-day mortality of the critical patients was reported to be approximately 61.5% [8]. It is well known that most patients with COVID-19 have fever along with nonspecific respiratory symptoms, such as cough, expectoration and dyspnea. The prevalence of extrapulmonary symptoms often arise from digestive system, so many patients initially present with diarrhea, nausea and vomiting [9]. Unfortunately, no specific therapeutic interventions exist so far, thus, supportive care remains the cornerstone for managing patients. A novel vaccine of COVID-19 is being conducted for clinical trials, which might make a great progress for the further treatments.

Most cases of COVID-19 are mild, however, patients with severe disease can quickly progress to multiple organ dysfunction syndrome (MODS) and even death. Approximately 5% patients develop critical illness requiring an intensive care unit (ICU) admission [10]. Those admitted to the  ICU were found to have higher concentrations of pro-inflammatory cytokines as well as T-helper-2 (Th2) cytokines suppressing inflammatory responses [11]. Aberrant immune-inflammatory storm and anti-inflammatory syndrome may play an important role in the disease progression [12]. Recently, investigators have found the rising of neutrophils as well as the decreasing of lymphocytes can be used as an indicator of disease progression. Other immune-inflammatory biomarkers, such as higher levels of interleukin-6 (IL-6), C-reaction protein (CRP) and Procalcitonin (PCT) were independent risk factors for assessing the severity of COVID-19 in patients [12, 13]. However, to predict the disease severity through potential risk factors at an early stage is still an unsolved problem, especially for the most serious area of Wuhan in China. Therefore, we performed a retrospective study to evaluate the diagnostic value of clinical features and laboratory markers to discriminate between severe and non-severe COVID-19 disease among adults presenting to hospital.

## Methods

### Data collection

The Tongji Hospital Affiliated to Tongji Medical College of Huazhong University of Science and Technology in Wuhan was designated to treat COVID-19. Nasal swab or pharyngeal swab specimens were collected from all suspected SARS-CoV-2 infection patients either in the emergency room or during hospitalization. A confirmed case of COVID-19 was defined as positive result by real-time reverse transcription polymerase chain reaction (RT-PCR) method, carried out according to a validated protocol. A consecutive sample of 187 hospitalized patients, with confirmed COVID-19 from January 27th to March 8th, 2020 were enrolled. During in-hospital period, patients were mainly treated with supportive cares. All patients received an antivirotic treatment, arbidol, directing at the etiology. The majority of patients accepted antibiotic therapy, including moxifloxacin and Kanamycin, to improve their anti-infection ability. For severe patients, fluid resuscitation, nutritional support, and endotracheal intubation were performed if necessary. A total of 77 cases died at the end of our deadline. Study participants were classified as severe or non-severe COVID-19 disease, based on criteria from the seventh edition of the Chinese National Health Commission disease classification system. Severe disease was based on the presence of the following criteria: First, shortness of breath, respiratory rate (RR) ≥ 30 times/min; Second, oxygen saturation ≤ 93% on room air at rest; Third, partial pressure of arterial oxygen (PaO_2_)/oxygen concentration (FiO_2_) ≤ 300 mmHg; Fourth, pulmonary imaging recognized obvious lesion progression > 50% within 24–48 h [12, 13].

This study was approved by the Human Research Ethics Committee of the Tongji Hospital (TJ-IRB20200225) and written informed consents were obtained from all participants.

### Clinical symptoms and laboratory examinations

Demographic characteristics, symptoms, underlying comorbidities, and laboratory results were obtained from electronic medical records. The clinical symptoms included fever, cough, expectoration, chest distress, myalgia, fatigue, diarrhea, and nausea. Venous blood samples were collected on admission and were analyzed within 24-h. The results of blood, sputum and catheter cultures were obtained when available.

Routine blood tests were detected by HF-3000 hematology analyzer (HLIFE, China). Plasma inflammatory parameters, such as C-reaction protein and Procalcitonin, were tested by the Dry Fluorescent Immunoassay (SKY-300, China). Serum cytokines were measured by enzyme linked immunosorbent assay (ELISA) kit. The baseline laboratory examinations were performed within 3 days after admission.

### Statistical analysis

Continuous data were presented as a mean ± standard deviation (mean ± SD), categorical data were presented as frequencies and percentages. Differences in values between the severe and non-severe groups were compared using Student’s T-tests, or Chi-squared test, or Fisher’s exact, where appropriate. A multivariate logistic regression model was used to identify the best predictors of severe COVID-19. A liberal *p*-value of < 0.2 was used to include all potential predictors identified from univariate analysis. Receiver operator characteristics (ROC) curves and area under curve (AUC) for univariate comparison of predictors of severe COVID-19 disease  were also presented. Correlations between different variables were determined by Spearman rank correlation analysis. All levels of statistical significance were set at 0.05. All data management and analysis were performed using SPSS 22.0 and Graphpad Prism 6.0.

## Results

### Demographic characteristics of patients with COVID-19

Characteristics of study participants, based on severity of COVID-19, are presented in Table 1. Among the 187 patients, 144 cases (77%) were classified into severe COVID-19 disease, and 111 (59%) were male. The severe group were older in age (62.6 ± 1.10 vs 57.7 ± 2.28, *p* = 0.04). The most common symptoms of all patients were fever (90%), cough (83%) and expectoration (54%). A higher ratio of cough (98% vs 78%, *p* = 0.001) and expectoration (87% vs 44%, *p* < 0.001) was observed in the non-severe group, compared to the severe group. Other common symptoms such as myalgia (37% vs 15%, *p* = 0.002), and fatigue (42% vs 22%, *p* = 0.018), were also more common in the non-severe group. No difference in the rates of underlying disease or medical history was observed between the severe and non-severe COVID-19 groups (Table 1). Compared with the non-severe COVID-19 group, the duration of the infection was significantly increased in severe group (p = 0.011) (Table 1).

**Table 1:** Demographic characteristics of patients with COVID-19

Variables	All patients (n = 187)	Non-severe group (n = 43)	Severe group (n = 144)	p value
Age (years)	61.53 ± 1.675	57.67 ± 2.275	62.55 ± 1.098	**0.0404**
Gender				
Male	111 (59.36)	28 (65.12)	83 (57.64)	0.4795
Female	76 (40.64)	15 (34.88)	61 (42.36)	
Symptoms				
Fever	169 (90.37)	42 (97.67)	127 (88.19)	0.0784
Cough	154 (82.35)	42 (97.67)	112 (77.78)	0.0012
Expectoration	101 (54.01)	37 (86.05)	64 (44.44)	< 0.0001
Chest distress	77 (41.18)	13 (30.23)	64 (44.44)	0.1134
Myalgia	37 (19.79)	16 (37.21)	21 (14.58)	0.0020
Fatigue	50 (26.74)	18 (41.86)	32 (22.22)	0.0175
Diarrhea	48 (25.67)	9 (20.93)	39 (27.08)	0.5510
Nausea	18 (9.63)	6 (13.95)	12 (8.33)	0.3746
Time interval (days)				
On admission	2 (0–4)	2 (0–3)	2 (1–4)	0.7675
Discharge	24 (14–39)	20 (14–26)	29 (22–39)	0.0113
Underlying diseases				
Hypertension	89 (47.59)	18 (41.86)	71 (49.31)	0.4868
Diabetes	33 (17.65)	5 (11.63)	28 (19.44)	0.3611
Cardiovascular disease	21 (11.23)	6 (13.95)	15 (10.42)	0.5825
Chronic lung disease	12 (6.42)	2 (4.65)	10 (6.94)	0.7365
Chronic hepatic disease	3 (1.60)	1 (2.33)	2 (1.39)	0.5456
Chronic kidney disease	3 (1.60)	1 (2.33)	2 (1.39)	0.5456
Past history				
Smoking	8 (4.28)	1 (2.33)	7 (4.86)	0.6842
Drinking	5 (2.67)	2 (4.65)	3 (2.08)	0.3245

### Laboratory markers

Baseline laboratory results for severe and non-severe patients are presented in Table 2. Higher levels of inflammatory markers were associated with severe disease: leukocyte, *p* = 0.001; neutrophils, *p* < 0.001; C-reaction protein (CRP), p = 0.021; interleukin-6 (IL-6), *p* = 0.031; and, interleukin-8 (IL-8), *p* =  0.007. Platelet counts were lower in the severe group compared to the non-severe (*p* < 0.001). Other inflammatory cytokines, including interleukin-10 (IL-10) and tumor necrosis factor-a (TNF-a), were no different, beyond chance, between the two groups (all *p*-values > 0.05).

**Table 2:** Laboratory markers of patients with COVID-19

Variables	All patients (n = 187)	Non-severe group (n = 43)	Severe group (n = 144)	p value
WBC count (× 10^9^)	8.62 ± 0.40	5.59 ± 0.27	9.50 ± 0.51	0.001
Neutrophils (× 10^9^)	6.45 ± 0.37	3.47 ± 0.22	7.32 ± 0.52	< 0.001
Lymphocyte (× 10^9^)	1.55 ± 0.18	2.43 ± 0.11	1.30 ± 0.24	0.157
Hemoglobin (g/L)	118 ± 2.45	123 ± 2.91	117 ± 1.99	0.096
Platelet (× 10^9^)	180 ± 10.9	240 ± 12.6	163 ± 9.23	< 0.001
C-reaction protein (mg/L)	66.4 ± 8.48	22.4 ± 9.86	73.6 ± 7.14	0.021
Procalcitonin (ng/mL)	1.45 ± 0.84	1.40 ± 1.22	1.46 ± 0.54	0.961
Interleukin-6 (pg/mL)	66.02 ± 3.82	6.62 ± 3.15	78.52 ± 4.49	0.031
Interleukin-8 (pg/mL)	37.84 ± 4.50	8.96 ± 2.85	43.98 ± 6.12	0.007
Interleukin-10 (pg/mL)	12.2 ± 2.13	5.8 ± 1.56	13.5 ± 2.70	0.056
Tumor necrosis factor-a (pg/mL)	13.0 ± 1.72	8.3 ± 1.33	13.9 ± 1.93	0.148

### Logistic regression analysis for the severe COVID-19

A multivariate logistic regression analysis was performed to identify predictors of severe COVID-19. Using a *p*-value < 0.2, as the cut-off for inclusion, age, hemoglobin, platelets, and inflammatory associated markers were included into the final analysis. The results demonstrated that only neutrophils (*p*-values = 0.027, odd ratio (OR) = 1.541, confidence interval (95% CI) 1.049–2.262) and platelets (*p*-values = 0.004, OR = 0.890, confidence interval (95% CI) 0.884–0.997) were independent risk factors for identifying severe COVID-19 (Table 3).

**Table 3:** Logistic regression analysis of variables for the severe COVID-19

Variables	Univariate analysis p value	OR (odd ratio)	Multivariate analysis p value	OR (odd ratio)
Age	0.0404	1.031	0.793	0.994
WBC count	0.001	1.220	0.890	1.009
Neutrophils	< 0.001	1.325	0.027	1.541
Lymphocyte	0.157	0.940	0.123	0.992
Hemoglobin	0.096	0.987	0.766	0.996
Platelet	< 0.001	0.993	0.004	0.890
C-reaction protein	0.021	1.014	0.791	0.998
Interleukin-6	0.031	1.040	0.846	1.006
Interleukin-8	0.007	1.105	0.153	1.180
Interleukin-10	0.056	1.210	0.090	0.741
Tumor necrosis factor-a	0.148	1.059	0.723	1.062

### ROC curves for predicting the severe COVID-19

**Fig. 1:**
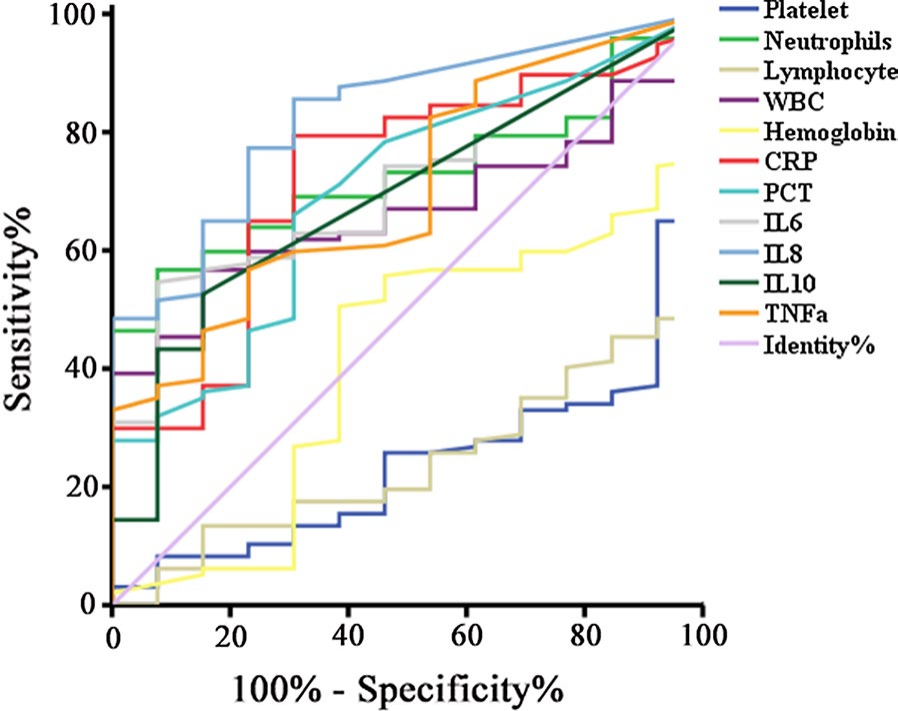
ROC curves for predicting the severe COVID-19

**Fig. 2:**
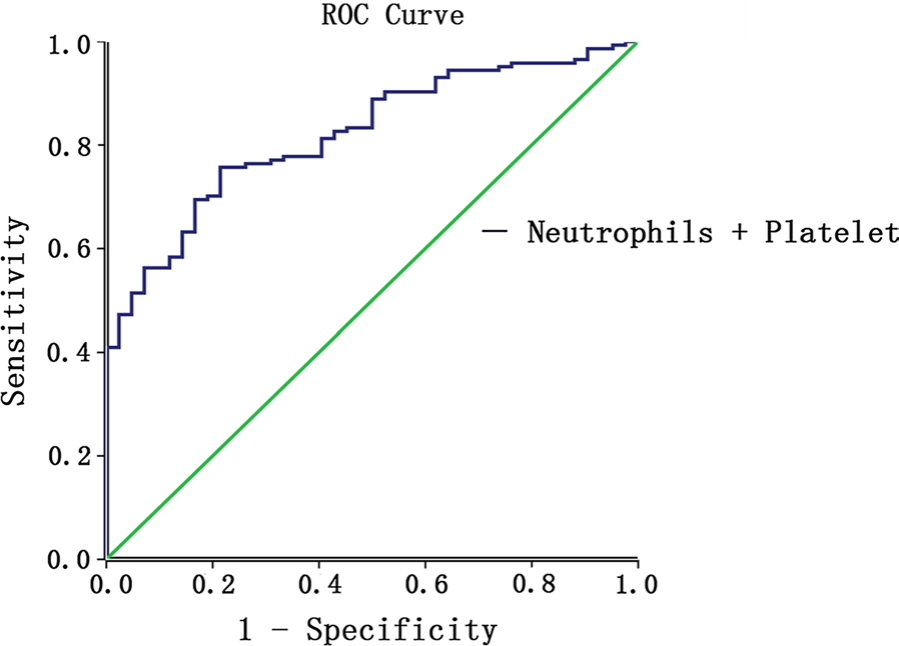
A combined ROC curve of neutrophils and platelet for severe COVID-19 prediction

ROC curves and associated AUC are presented in Figs. 1, 2. AUC values were 0.722 (CRP), 0.697 (PCT), 0.706 (IL-6), 0.828 (IL-8), 0.689 (IL-10) and 0.704 (TNF-a). This suggested a reasonable prediction for the COVID-19 severity by the above immune-inflammatory biomarkers. What’s more, lower levels of lymphocytes and platelets could also indicate severe disease, since AUC values were 0.761 and 0.764, respectively. Besides, since neutrophils and platelet were meaningful in multivariate analysis, a combined diagnosis of severity in ROC analysis was conducted. The combined AUC was as high as 0.821, which presented an excellent prediction (Table 4).

**Table 4:** ROC curve of risk model and laboratory parameters for the severity of COVID-19

Variables	AUROC	Std. error	p value	Asymptotic 95% confidence interval	
				Lower bound	Upper bound
WBC	0.618	0.057	0.169	0.506	0.729
Neutrophils	0.681	0.056	0.034	0.572	0.790
Lymphocyte	0.761	0.052	0.002	0.138	0.341
Hemoglobin	0.408	0.079	0.281	0.252	0.563
Platelet	0.764	0.055	0.002	0.129	0.344
C-reaction protein	0.722	0.071	0.010	0.584	0.860
Procalcitonin	0.697	0.072	0.022	0.555	0.838
Interleukin-6	0.706	0.061	0.016	0.587	0.825
Interleukin-8	0.828	0.052	< 0.001	0.725	0.930
Interleukin-10	0.689	0.069	0.027	0.553	0.825
Tumor necrosis factor-a	0.704	0.069	0.017	0.569	0.839
Neutrophils + platelet	0.821	0.032	< 0.001	0.758	0.883

*ROC* receiver operator characteristics, *AUROC* area under receivers operator characteristic curve

### Correlations between clinical serum markers

**Fig. 3:**
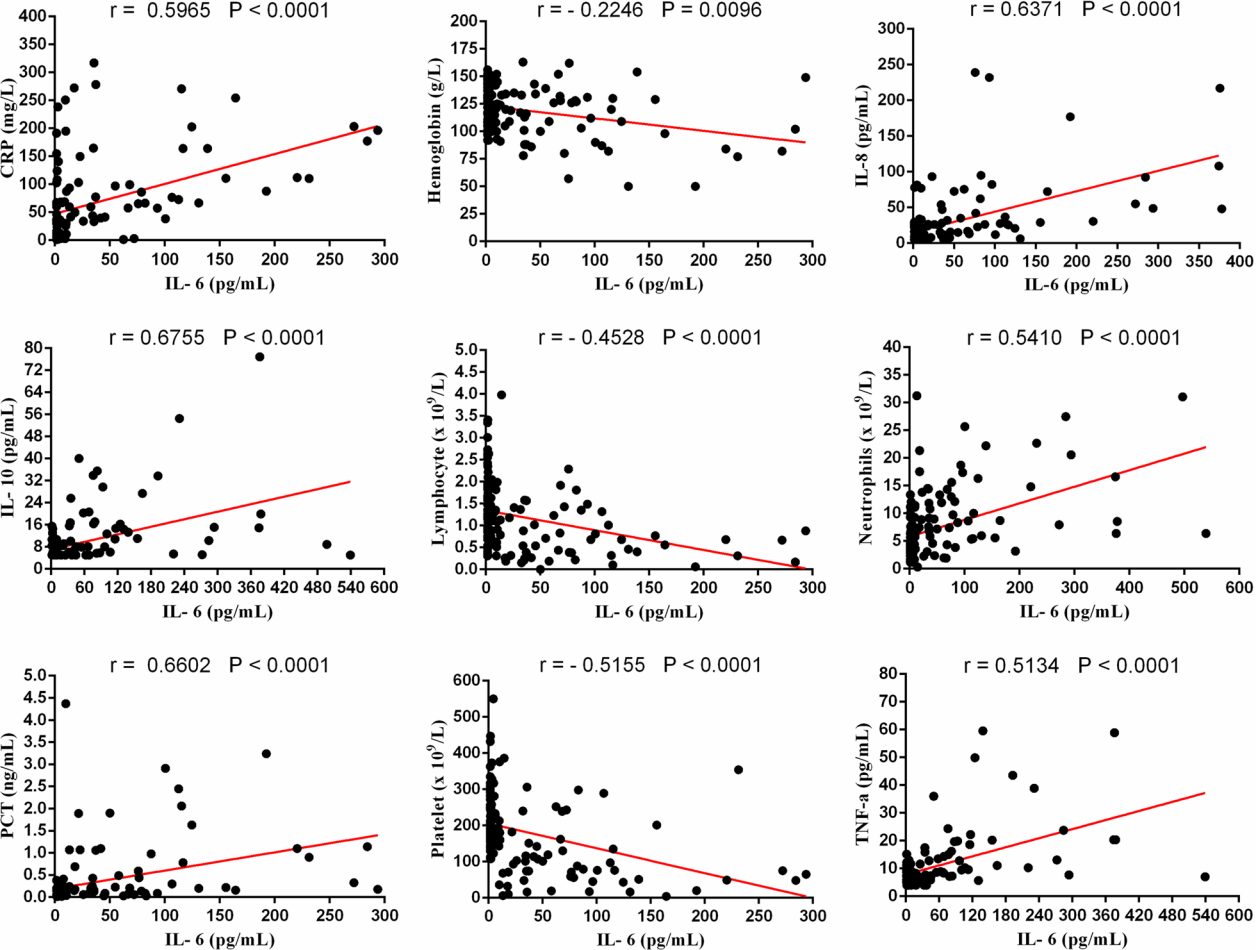
Correlations between IL-6 and neutrophils, lymphocyte, hemoglobin, platelet, CRP, IL-8, IL-10, TNF-a and PCT in patients with COVID-19

**Fig. 4:**
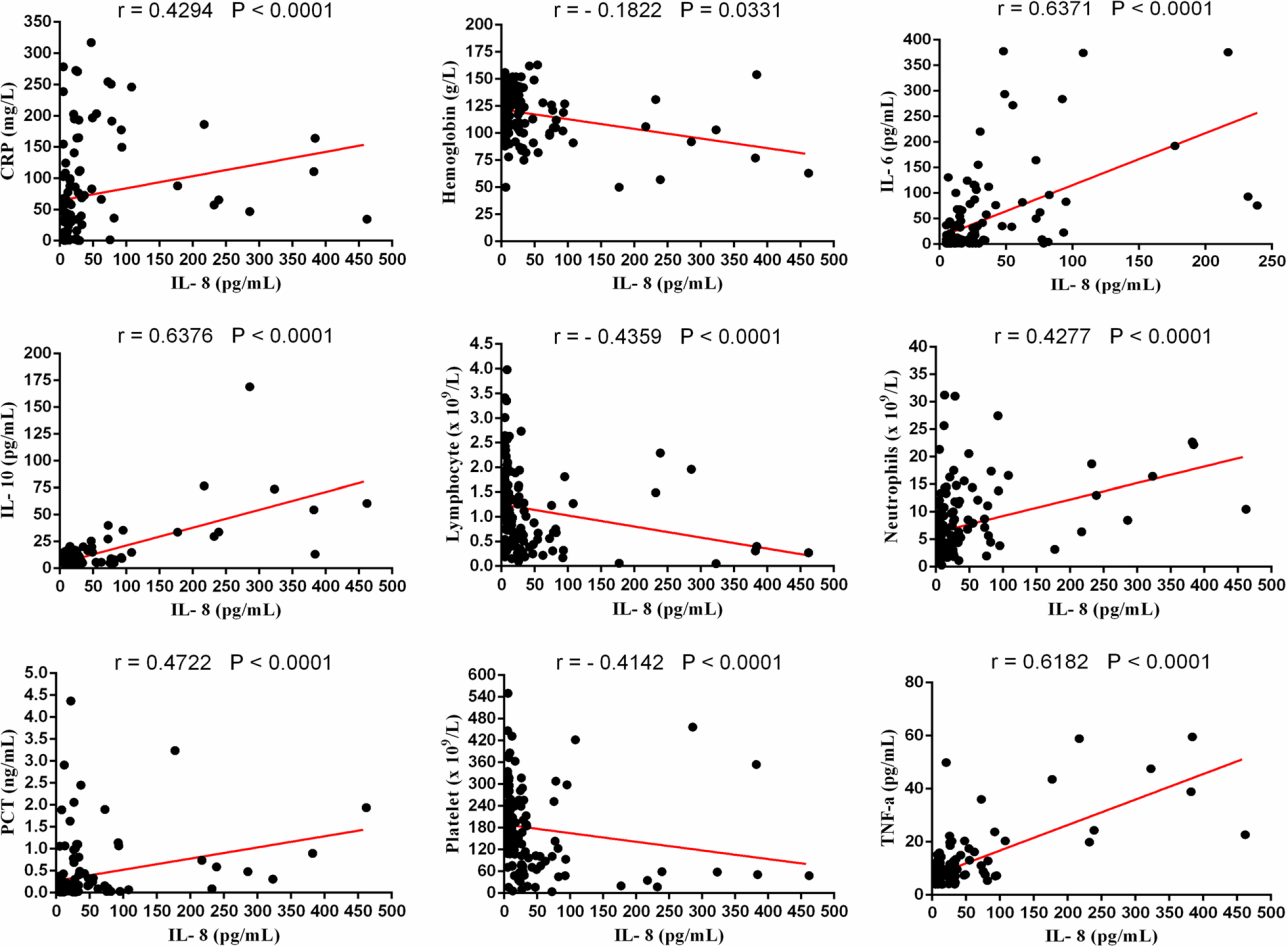
Correlations between IL-8 and neutrophils, lymphocyte, hemoglobin, platelet, CRP, IL-6, IL-10, TNF-a and PCT in patients with COVID-19

**Fig. 5:**
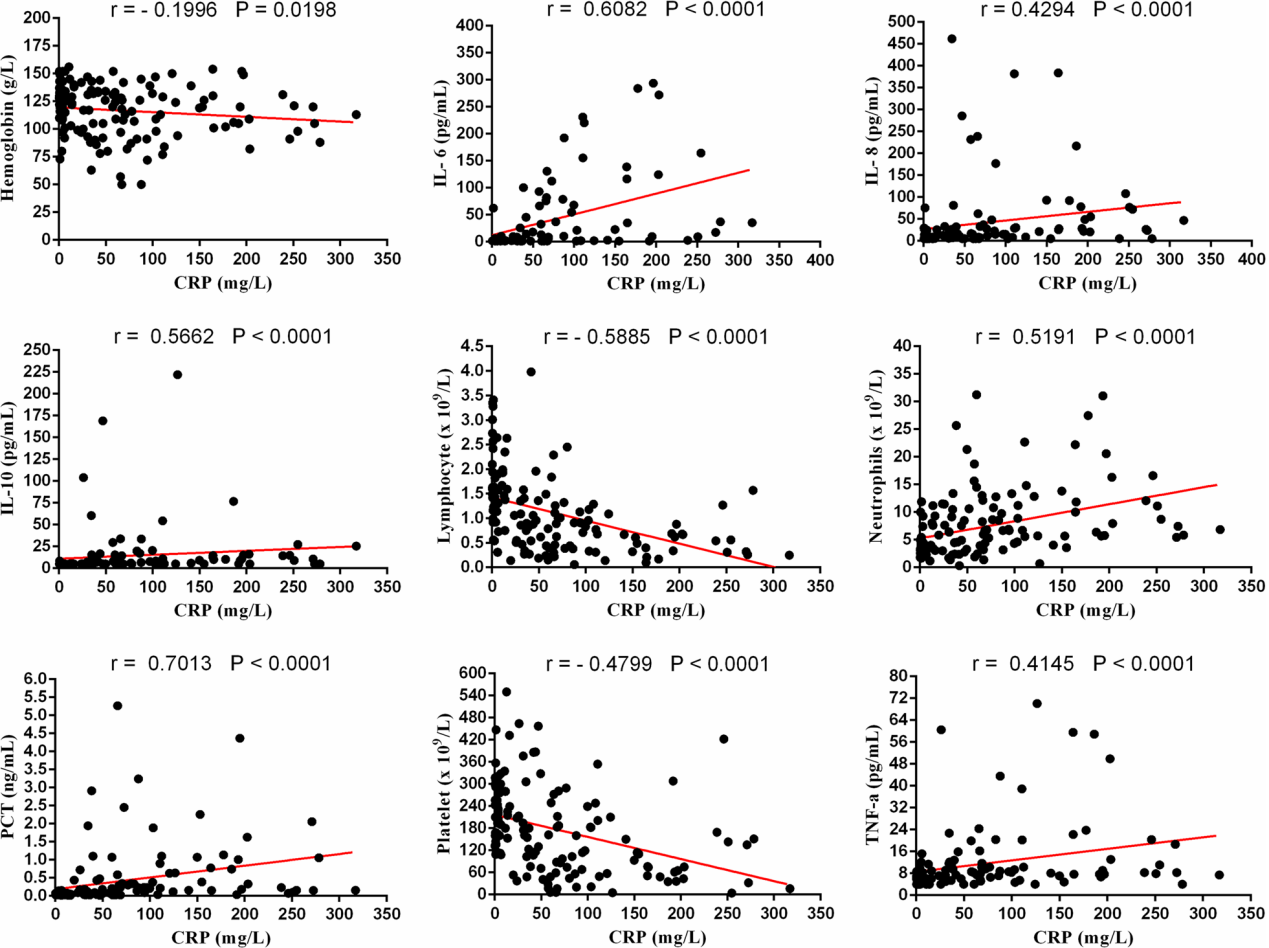
Correlations between CRP and neutrophils, lymphocyte, hemoglobin, platelet, IL-6, IL-8, IL-10, TNF-a and PCT in patients with COVID-19

**Fig. 6:**
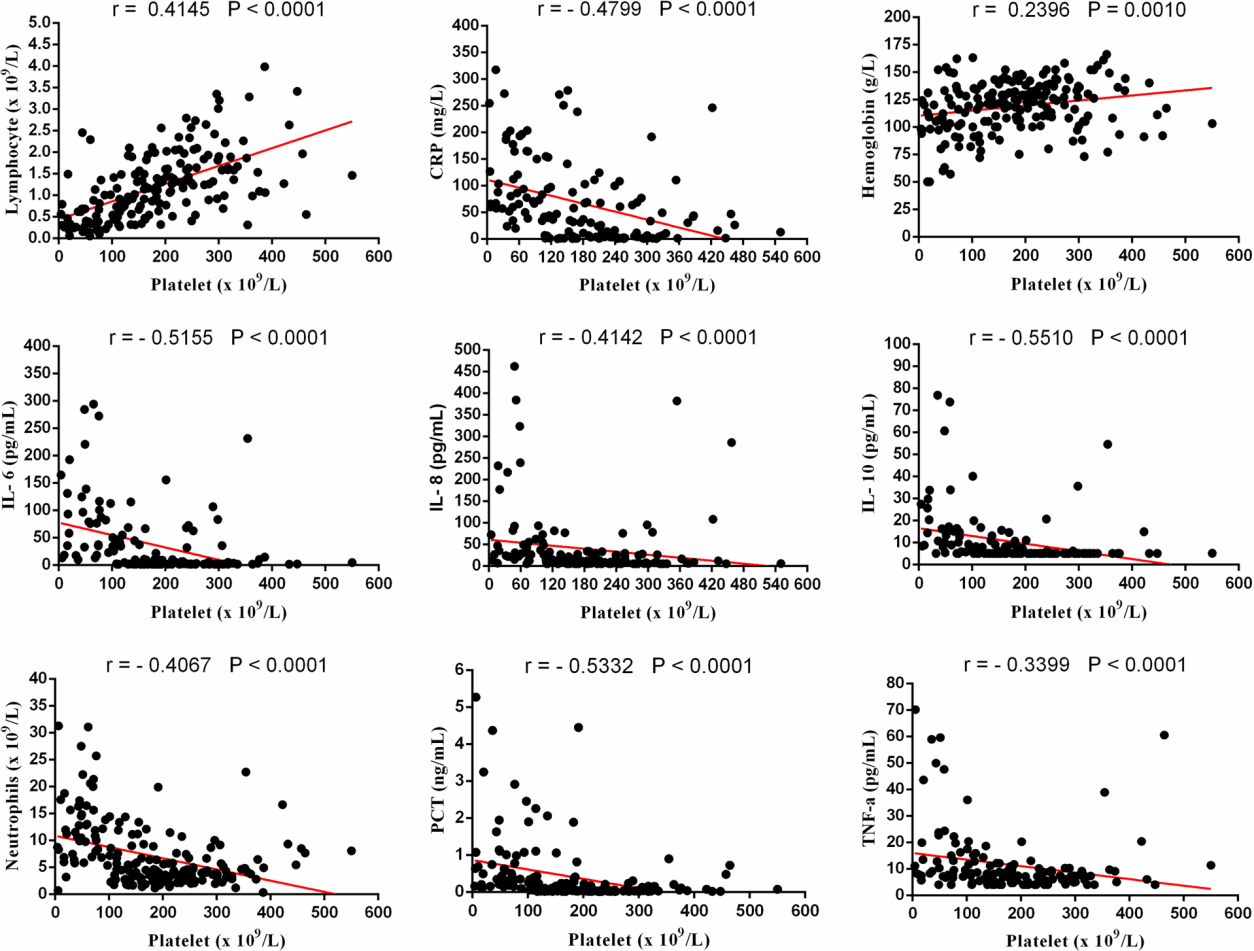
Correlations between platelet and neutrophils, lymphocyte, hemoglobin, IL-6, CRP, IL-8, IL-10, TNF-a and PCT in patients with COVID-19

Since the IL-6, IL-8, CRP and platelet markers were significant predictors for COVID-19 severity, we further analyzed their correlations with other variables. There was a positive correlation between the baseline IL-6 and neutrophils (r = 0.5410, *p* < 0.0001), IL-8 (r = 0.6371, *p* < 0.0001), IL-10 (r = 0.6755, *p* < 0.0001), TNF-a (r = 0.5134, *p* < 0.0001), CRP (r = 0.5965, *p* < 0.0001) and PCT (r = 0.6602, *p* < 0.0001). Meanwhile, it was negatively correlated with lymphocyte (r = − 0.4528, *p* < 0.0001), hemoglobin (r = − 0.2246, *p* = 0.0096) and platelet counts (r = − 0.5155, *p* < 0.0001) (Fig. 3). The similar result also appeared to the other two immune-inflammatory biomarkers (IL-8 and CRP). The IL-8 was found to be positively related to neutrophils (r = 0.4277, *p* < 0.0001), IL-6 (r = 0.6371, *p* < 0.0001), IL-10 (r = 0.6376, *p* < 0.0001), TNF-a (r = 0.6182, *p* < 0.0001), CRP (r = 0.4294, *p* < 0.0001) and PCT (r = 0.4722, *p* < 0.0001), while negatively related to lymphocyte (r = − 0.4359, *p* < 0.0001), hemoglobin (r = − 0.1822, *p* = 0.0331) and platelet counts (r = − 0.4142, *p* < 0.0001) (Fig. 4). Additionally, the baseline of CRP was positively correlated with neutrophils (r = 0.5191, *p* < 0.0001), IL-6 (r = 0.6082, *p* < 0.0001), IL-8 (r = 0.4294, *p* < 0.0001), IL-10 (r = 0.5662, *p* < 0.0001), TNF-a (r = 0.4145, *p* < 0.0001) and PCT (r = 0.7013, *p* < 0.0001), whereas negatively correlated with lymphocyte (r = − 0.5885, *p* < 0.0001), hemoglobin (r = − 0.1996, *p* = 0.0198) and platelet counts (r = − 0.4799, *p* < 0.0001) (Fig. 5). Besides, it  was interesting to note a common negative relationship between platelet counts and other parameters, including neutrophils (r = − 0.4067, *p* < 0.0001), IL-6 (r = − 0.5155, *p* < 0.0001), IL-8 (r = − 0.4142, *p* < 0.0001), IL-10 (r = − 0.5510, *p* < 0.0001), TNF-a (r = − 0.3399, *p* < 0.0001), CRP (r = − 0.4799, *p* < 0.0001) and PCT (r = − 0.5332, *p* < 0.0001). Meanwhile, platelet count was positively related to lymphocyte (r = 0.4145, *p* < 0.0001) and hemoglobin (r = 0.2396, *p* = 0.0010) (Fig. 6).

### Different cultures and the severity of COVID-19

**Fig. 7:**
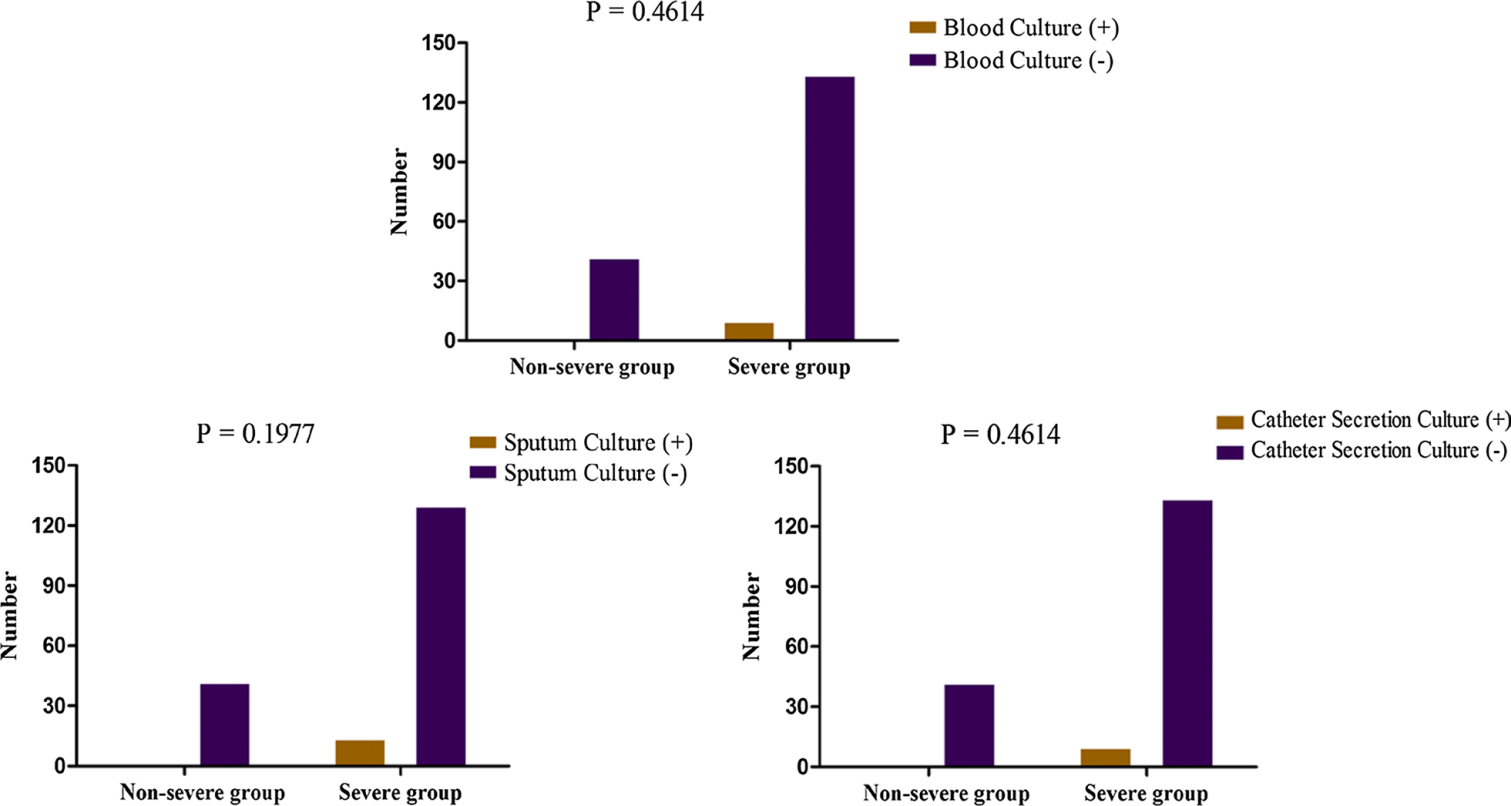
Different cultures and the severity of COVID-19

In this study, positive blood cultures and sputum cultures were not associated with severe disease (*p* = 0.461 and 0.198, respectively). Catheter secretion cultures from the hydrothorax, or ascites were also not associated with more severe COVID-19 disease (*p* = 0.461) (Fig. 7).

## Discussion

This study has been able to identify characteristics associated with severe COVID-19 disease among adults presenting to hospital. Most importantly, higher levels of inflammatory markers and lower platelet levels have been shown to be strongly associated with more severe disease. It was also shown that the blood, sputum or catheter secretion culture did not show any prediction for disease severity.

The association between increasing age and more severe COVID-19 found in our study, is consistent with previous reports by Tan et al. [14] and Xu et al. [15]. On average patients who did not survive to hospital discharge were 20 years older than those surviving to discharge [15]. The overall prognosis of older patients was worse than that of young or middle-aged patients might be attributed to their poor state of malnutrition or underling complications [16]. In our study, symptoms of cough, expectoration, myalgia and fatigue were significantly higher in the non-severe group. As severe patients  might have already passed this symptomatic process into a multiple organ dysfunction stage, could explain the lower incidence of overt symptoms in this group compared to the non-severe group. In contrast to other reports, this study failed to show any difference in the rates of underlying diseases or past medical history between the severe and non-severe COVID-19 groups. This conflicts with a systematic review by Zheng et al. [17] that reported higher rates of underlying diseases such as hypertension, diabetes, cardiovascular disease and respiratory disease among critically ill patients.

In terms of laboratory results, several studies have reported increased neutrophil counts and decreased lymphocyte counts as a strong predictor for severe COVID-19 disease. For example, Wan et al. [2] reported the leukocyte counts of most patients with COVID-19 were in a normal range, but the classified neutrophil counts were significantly higher in the severe group. Zhang et al. [1] reported lymphocyte counts were lower among deteriorating patients compared to those in being discharged. In our study, both neutrophil and lymphocyte counts were shown to have excellent predictive ability to identify severe COVID-19 disease, which was in agreement with the typical progress of virus infection. Another important finding in our study was related to platelet levels, a lower level of platelets was identified as an independent risk factor for severe COVID-19, which was in agreement with the study published by Zhe Zhu et al. [8]. As we know, platelets are small (2–4 µm in diameter) anucleated cells derived from megakaryocytes in bone marrow, and are responsible for maintaining the integrity of the vasculature. In COVID-19 subjects, platelets were hyperactivated, with aggregation occurring at suboptimal thrombin concentrations, so the circulation level were significantly decreased [18]. What’s more, the severe cases experienced higher viral infection and mechanical ventilation, which led to endothelial injury, platelet aggregation and megakaryocyte reduction, and as a result, the platelet production decreased and the consumption increased [19]. In turn, a common negative correlation was found between platelet and other parameters increasing along with the severity of disease.

With disease progression, vigorous pro-/anti-inflammatory responses to the virus induced apoptosis in lung epithelial and endothelial cells. Inflammatory mediators play an important role in the pathogenesis of severe disease. CRP as an acute-phase protein that stimulated by the release of apoptosis cells, could indicate the severity of inflammation. Prior studies have revealed that CRP on admission could be a predictive factor for progressive respiratory failure in SARS-CoV as well as MERS-CoV infected patients [20]. A recent study by Aggarwal et al. demonstrated that the CRP level showed no obvious difference between critically ill COVID-19 patients and those with mild disease [21]. However, Wang [22] published their research about CRP, showing that an early stage of COVID-19 CRP levels  was positively correlated with lung lesions and could reflect disease severity. Our study showed a similar result with the latter, confirming its role in predicting the severity COVID-19. As a quality improvement project, PCT, a kind of procalcitonin without hormone activity and the precursor of calcitonin [13], was introduced as an antibiotic stewardship tool, also reflecting the inflammatory response. A meta-analysis concluded that PCT > 0.5 ng/mL  was associated with increased risk of COVID-19 critical illness especially when the leukocyte  was initially normal or reduced [23]. In our study, the AUC of PCT was 0.697, providing a reasonable discrimination between individuals with severe and non-severe COVID-19. In addition, these two inflammatory associated factors (CRP and PCT)  were significantly correlated with other cytokines, indicating the fact that excess inflammatory responses deteriorated the normal physiological function in viral infection, making the disease worse in progress.

Cytokines are small protein molecules that play an immunomodulating function to maintain the body homeostasis. The auto-amplifying of pro-inflammatory cytokines, such as IL-6, IL-8, and TNF-a, can result in a dramatic deterioration for a host, often referred to a “cytokine storm”. This contrasts with an excessive secretion of anti-inflammatory cytokines (IL-10), which might lead to the persistent immunoparalysis and high risk of secondary infection. Several studies have attempted to explore the relationship between inflammatory cytokines and COVID-19. Zhu et al. [12] found that high levels of IL-6 was an independent risk factor for severe COVID-19, and  it had a potential value in the monitoring of the disease course in severe cases. Zhang et al. [1] investigated a number of factors related to disease progression among hospitalized patients with COVID-19, also  discovered that IL-6 and IL-10 were elevated among those with worse outcomes. Consistent with previous studies, our study also showed that IL-6 and IL-8 were higher among severe versus non-severe patients. Overall, our study showed that high levels of immune-inflammatory markers (IL-6, IL-8, IL-10 and TNF-a) were all strong predictors of severe COVID-19  by ROC analysis. IL-6 is a multifunctional cytokine that transmits cell signaling and regulates immune cells. It not only plays a key role in cytokine storm, but induces a variety of acute-phase protein, such as CRP, complement components and so on. A recent study confirmed the role of IL-6 in severe COVID-19 cases by immune analysis, suggesting that Th1 cells and monocytes could express high level of IL-6, resulting in deteriorating the tracheobronchial epithelial cells and aggravating the disease [12]. IL-8 has a potential function in recruitment and activation of neutrophils, and combines with the special surface antibody releasing large numbers of reactive oxygen radicals to aggravate the lung injury. Therefore IL-8 commonly elevates in patients with critical disease, and presents a state of inflammation and organ damage [24]. Consequently, there is no doubt that IL-6 and IL-8 have a positive relationship with other immune-inflammatory markers. TNF-a, another important inflammatory cytokine, acts as an early and sensitive mediator in cytokine storm. Once the viral infection induces the inflammatory defense response, TNF-a could be stimulated by activated neutrophils and lymphocyte and exerted a synergistic effect in the secretion of other cytokines [25]. In contrast to the mentioned pro-inflammatory cytokines, IL-10, as a classical anti-inflammatory cytokine, takes part in maintaining the immune system balance. Since critical patients present a higher level of inflammatory response, regulatory T cells (Tregs) are induced to inhabit the over activation immune response through producing anti-inflammatory cytokines, such as IL-10, transcription growth factor-β (TGF-β) [26]. As a result, IL-10 also shows a positive correlation with other immune-inflammatory parameters.

In order to clarify the inner correlations between different serum markers, four indicators (IL-6, IL-8, CRP and Platelet) were selected for further correlation analysis. These biomarkers were chosen because they presented a significant prediction for COVID-19 severity in both univariate analysis and ROC analysis. The current result suggested that pro-inflammatory biomarkers, such as IL-6, IL-8 and CRP, showed a major positive correlation with other indicators, except for lymphocyte, hemoglobin and platelet. While platelet presented a central negative correlation, along with the progress of inflammatory response.

Lastly, few researchers have drawn on any structured research into the association between various cultures and severity of COVID-19. Our study included an analysis of a large number of varying blood, sputum and catheter cultures, which were not associated with more severe COVID-19 disease. The reason might be the lower incidence of positive secretion cultures in our hospital.

Currently, researchers are reporting some new indicators for severe COVID-19, which might be the next step for our research group. For example, Yang et al.  [27] showed that serum apolipoprotein A 1 served as an indicator to reflect the severity of COVID-19. Gómez-Pastora et al. [28] examined the level of ferritin in COVID-19 patients, and confirmed hyperferritinemia in the critically ill, but failed to reveal whether it was the product of inflammation or a pathogenic mediator. Critical patients often experience the respiratory failure in the late stage, so mechanical ventilation is a necessary method to relieve symptoms. At this point, Poggiali et al. [29] presented that Lactate dehydrogenase (LDH) was a predictor of respiratory failure in severe cases. Lastly, adaptive immune response is thought to protect from acquiring SARS-CoV-2 infection, of which neutralizing antibodies (NtAb) seemingly play a major role, such as NtAb50. However, Gozalbo-Rovira et al. [30] found very weak correlations between NtAb50 and COVID-19 severity.

The results of our study need to be considered in the context of potential limitations. Firstly, this is a single-center and retrospective study. Large prospective studies, over an extended period of time, is needed to verify our results. Secondly, the inflammatory response associated with cytokines IL-10 and TNF-a, although showing promise in ROC AUC analysis to predict severe disease, did not reach statistical significance in logistic regression analysis,  this may represent Type II error. Lastly, another potential limitation is that we failed to show any relationship between various cultures and COVID-19 severity, which may be due to our low positive detection rate, and would need to be confirmed by larger studies in the future.

## Conclusions

A retrospective study was conducted to evaluate the diagnostic value of clinical features and laboratory markers to discriminate between severe and non-severe COVID-19 disease. Higher serum levels of inflammatory markers appear to be strong predictors of severe COVID-19, various serum biomarkers coordinate with each other to promote the disease progress. The present study suggested that high leucocyte and low platelets counts were independent predictive markers of severity of COVID-19.

## Data Availability

The datasets used and/or analysed during the current study are available from the corresponding author on reasonable request.

## Abbreviations

COVID-19: Coronavirus disease 2019
SARS: Severe acute respiratory syndrome
MERS: Middle east respiratory syndrome
SARS-CoV-2: Severe acute respiratory syndrome coronavirus type 2
MODS: Multiple organ dysfunction syndrome
ICU: Intensive care unit
IL-6: Interleukin-6
CRP: C-reaction protein
PCT: Procalcitonin
ROC: Receiver operator characteristics
AUC: Area under curve
IL-8: Interleukin-8
IL-10: Interleukin-10
TNF-a: Tumor necrosis factor-a
OR: Odd ratio
95% CI: Confidence interval
AUROC: Area under receivers operator characteristic curve
